# The relationship between maximal lactate accumulation rate and sprint performance parameters in male competitive swimmers

**DOI:** 10.3389/fspor.2024.1483659

**Published:** 2024-10-22

**Authors:** Yasuo Sengoku, Anna Shinno, Jaewoo Kim, Kenta Homoto, Yusaku Nakazono, Takaaki Tsunokawa, Norimasa Hirai, Ayaka Nobue, Masaki Ishikawa

**Affiliations:** ^1^ Institute of Health and Sport Sciences, University of Tsukuba, Tsukuba, Japan; ^2^ Graduate School of Comprehensive Human Sciences, University of Tsukuba, Tsukuba, Japan; ^3^ Department of Sport Wellness Sciences, Japan Women's College of Physical Education, Tokyo, Japan; ^4^ Toyo University, Tokyo, Japan; ^5^ Morinomiya University of Medical Sciences, Osaka, Japan; ^6^ Osaka University of Health and Sport Sciences, Osaka, Japan

**Keywords:** anaerobic capacity, glycolytic power, blood lactate, load-velocity profile, front crawl swimming

## Abstract

This study aimed to examine the relationship between the maximal lactate accumulation rate (ċLa_max_) and sprint performance parameters in male competitive swimmers. Seventeen male competitive swimmers volunteered to perform a 20 m maximal front crawl sprint without pushing off the wall from a floating position. ċLa_max_ was determined by the 20-m sprint time and blood lactate measured before and after the 20 m sprint. For the sprint performance parameter, a 50 m time trial with the front crawl swimming stroke was conducted, and the times taken from 0 to 15 m, 15–25 m, 25–35 m, and 35–45 m were analyzed. A semi-tethered swimming test was conducted to investigate the load-velocity profile of each swimmer. From the load-velocity profile, theoretical maximal velocity (V_0_), maximal load (L_0_) and relative maximal load (rL_0_) were examined. The slope of the load-velocity profile was also determined. According to the results, ċLa_max_ correlated with 50 m front crawl performance (*r* = −.546, *p* < .05). Moreover, a higher ċLa_max_ was related to faster 0–35 m section time. Furthermore, ċLa_max_ correlated with L_0_ (*r* = .837, *p* < .01), rL_0_ (*r* = .820, *p* < .01), and load-velocity slope (*r* = .804, *p* < .01). ċLa_max_ is a good indicator of 50 m front crawl performance in male swimmers, and higher glycolytic power contributes to the faster time at the beginning of the sprint race. ċLa_max_ could also evaluate the ability of a swimmer to apply force to the water during high-intensity swimming.

## 1 Introduction

Competitive swimming events include different distances, from 50 m to 1,500 m. The energy required to swim these distances is supplied by the aerobic and anaerobic systems; the relative contributions of each energy system vary depending on the duration of each race (1). Therefore, improvements in both energy systems are essential to enhance swimming performance, and effective monitoring is key to assessing the impact of training and achieving successful performance (2).

Several reliable parameters are available to monitor aerobic capacity in swimmers, including lactate threshold (3, 4), onset of blood lactate accumulation (5), and critical velocity (4, 6). Measurement of maximal accumulated oxygen uptake (MAOD) is widely used to assess anaerobic capacity in sports and is also used in swimming (7–9). However, MAOD is a time-consuming procedure to measure, and it may not be a fully defensible method to determine anaerobic capacity (10). Therefore, alternative practical methods and indices have been explored (11, 12).

The maximal lactate accumulation rate (ċLa_max_) is also an index to access anaerobic capacity. ċLa_max_ is calculated by dividing the maximum difference in blood lactate (Bla) before and after a maximal sprint by the sprint time, which is reportedly a parameter to evaluate the athlete's glycolytic power (13). Due to its applicability in the training setting, ċLa_max_ regained popularity and was investigated recently in cycling (14, 15), running (16), and rowing exercises (17). In addition, Quittmann et al. (16) investigated ċLa_max_ during 100 m sprint running and reported that ċLa_max_ was significantly correlated with sprint performance parameters, suggesting that it can be used as a sport-specific field test. ċLa_max_ reportedly differs between handcycling and cycling (15) or cycling and running (18) even when investigated in the same individual, so it has been suggested that ċLa_max_ is an extremity and movement-specific parameter.

ċLa_max_ has also been investigated in swimming (19). Sperlich et al. (20) reported that ċLa_max_ increases with high intensity interval training and decreases with high volume training, and that ċLa_max_ is sensitive to detecting the change of anaerobic capacity in swimmers. Studies on the relationship between ċLa_max_ and sprint performance in swimming have yet to reach consensus. Mavroudi et al. (21) reported a significant correlation between 50 m sprint performance and ċLa_max_ which was investigated at that sprint. On the other hand, Kellar and Wahl (22) reported no significant correlation between ċLa_max_ assessed by a 20-s sprint test and the average velocity of a 50 m official race. Thus, it is still being determined whether ċLa_max_ is a genuinely valid measure that can assess anaerobic capacity in swimmers.

Mechanical power is one of the factors that determines maximal swimming velocity (23). Even a semi-tethered swimming test cannot evaluate pure propulsive power, Gonjo et al. (24) reported that the index of the load-velocity profile investigated by semi-tethered swimming can be used to evaluate sprint swimming performance. Their findings revealed that theoretical maximal velocity (V_0_), maximal load (L_0_), and load-velocity slope were significantly correlated with the mean velocity of a 50 m front crawl trial. Given the potential of these indices as indicators of sprinting ability, understanding the relationship between ċLa_max_ and these parameters could shed light on whether pure physiological capacity is related to sprinting ability in swimming. If glycolytic power is indeed related to sprint performance parameters, this approach would allow for a more comprehensive understanding of sprint swimming performance, combining both mechanical and physiological aspects.

The purpose of the present study was to investigate the relationship between ċLa_max_ and parameters related to sprint performance in male competitive swimmers. The primary hypothesis of this study was that ċLa_max_ negatively correlates with 50 m race time. Secondly, we hypothesized that ċLa_max_ positively correlates with parameters related to sprint performance (such as V_0_ and L_0_) estimated by the load-velocity profile. By addressing these research objectives, we provide valuable insights into the use of ċLa_max_ as an index of anaerobic capacity in competitive swimming.

## 2 Materials and methods

### 2.1 Participants

Seventeen well-trained male competitive swimmers participated in this study. The personal characteristics of the swimmers are shown in Table 1. Four swimmers specialized in short-distance front crawl, five in middle-distance front crawl, five in butterfly, two in backstroke, and one in individual medley. All swimmers (who were from the same university swimming team) had engaged in competitive swimming for over 6 years and competed at national-level championships. The World Aquatics point scoring of the swimmers’ personal best records of their major event was 751.8 ± 68.1 point. The participants were made fully aware of the risks, benefits, and stress factors of the study and gave their written consent to participate. In addition, the participants were instructed to refrain from caffeine intake within 12 h, and water was only allowed within 2 h of testing. This study received the formal approval of the Research Ethics Committee of the authors’ institution (No. 024-23) in compliance with the Declaration of Helsinki.

**Table 1:** Participants’ physical characteristics, major swimming style and performance level. The World Aquatics point scoring of the swimmers’ personal best records of their major event is demonstrated.

Subject	Body hieght	Body mass	Age	Major swimming style	World aquatics point
	(cm)	(kg)	(yrs)		
A	178.0	85.0	24	Butterfly	840
B	175.0	69.0	22	Front crawl (Short)	632
C	176.0	78.0	21	Backstroke	843
D	175.0	72.0	21	Front crawl (Short)	791
E	169.0	73.0	20	Butterfly	737
F	170.0	67.0	21	Butterfly	720
G	178.0	77.0	19	Front crawl (Short)	787
H	172.0	67.0	19	Front crawl (Short)	655
I	172.0	70.0	18	Butterfly	727
J	178.0	74.0	19	Backstroke	739
K	165.0	64.0	22	Front crawl (Middle)	601
L	170.0	69.0	20	Front crawl (Middle)	789
M	173.0	71.0	19	Front crawl (Middle)	776
N	177.0	73.0	19	Front crawl (Middle)	785
O	173.0	67.0	19	Front crawl (Middle)	797
P	175.0	69.0	18	Butterfly	787
Q	170.0	70.0	18	Individual Medlay	775
Ave	173.3	71.5	19.9		751.8
SD	3.7	5.0	1.7		68.1

### 2.2 Experimental procedure

All experiments were scheduled during the general and preparation period of the training season (2) and conducted in an indoor 50 m pool. Participants were instructed to conduct a self-selected warm-up mimicking their competition routine. This was followed by 5 min of passive rest while seated. The duration of the rest period was in accordance with previous research (15, 17, 18). After the passive rest, the participants performed a 20 m all-out front crawl sprint swim without pushing off the wall to evaluate ċLa_max_. In this trial, the participants were instructed to accelerate as fast as possible from a floating position at the 5 m point until they passed the 25 m point. Thereafter, a 50 m time trial and semi-tethered swimming test with a front crawl were conducted to investigate the sprint performance parameters. All experiments were separately conducted with minimum interval of 16 h and performed within one week.

### 2.3 ċLa_max_ investigation

Olbrecht (19) recommends that ċLa_max_ should be obtained and measured during a maximal sprint exercise of 10–15 s. In swimming, Mavroudi et al. (21) reported that ċLa_max_ values obtained in a 25 m all-out swim were significantly higher than those obtained in 35 m and 50 m all-out swims, shorter duration is appropriate for investigating ċLa_max_. Instead of a 25 m all-out swim, we adopted a 20 m sprint swim of front crawl starting from a floating position at the 5 m mark. As it is reported that ċLa_max_ is a limb and movement specific parameter (15, 18), this procedure eliminates the movement of the block start or wall push and the underwater phase and make it possible to execute the sprint exercise with front crawl swimming movement only. Our preliminary investigation confirmed that the duration of this swim sprint can last approximately 11 s, which falls within the range recommended by Olbrecht (19). ċLa_max_ was determined using Equation 1 (13, 19, 25):

(1) $$\dot{c}La_{\max}=(La_{\max}\text{–}La_{\mathrm{pre}})/(t_{\mathrm{sprint}}\text{–}t_{\mathrm{alac}})$$

where La_max_ is the highest Bla after the 20 m sprint, La_pre_ is the Bla measured during the passive rest before the sprint, *t*_sprint_ is the time to complete the 20 m sprint, and *t*_alac_ is the estimated time when energy is delivered by the alactic system.

Bla was measured using a portable analyzer (Lactate Pro2, LT-1730, Arkray, Kyoto, Japan). Considering the reliability and accuracy of the measurement device (26), each Bla was examined using two analyzers, and then the average value was used for analysis. Immediately after completing the 20 m sprint swim, participants were seated on the pool deck. An examiner started a stopwatch at the moment swimmer completed the 20 m sprint. Blood was sampled from the fingertips at precise one-minute intervals. The sampling and measurement process continued until the mean Bla value was lower than the previous measurement, indicating that the peak had been reached and passed. The timing of the blood sampling was consistent for all participants, ensuring standardized data collection across the study. The highest value was taken as La_max,_ and the time to reach La_max_ after the sprint (tLa_max_) was also examined. Two digital cameras (GC-IJ20B; Sports Sensing, Fukuoka, Japan) were set at 5 m and 25 m points to capture the timing of the passage of an LED marker attached to the participant's head through 5 m and 25 m marks of the pool. Their passing times and *t*_sprint_ were analyzed using 2-D motion analysis software (Frame Dias V, Q'sfix, Tokyo, Japan). Camera synchronization was achieved through an LED system (LED synchronizer, Q'sfix). *t*_alac_ is the period at the beginning of exercise for which no lactate production is assumed. In cycling exercise, *t*_alac_ can be determined as the time when power output decreased by 3.5% from peak power output directly measured during the maximal sprint (27, 28). However, as it is difficult to measure the power output during swimming exercise, a fixed standard value of 3 s was used for *t*_alac_ in this study according to previous research (13, 29). Eleven participants were retested with the same procedure within 4 days to confirm the reliability of the measurement data.

### 2.4 Sprint performance parameters

#### 2.4.1 50 m maximal trial

The 50 m time trial required participants to swim front crawl with maximum effort for the sprint performance parameter. After a self-selected warm-up on land and in water, the trial was performed starting from the starting block. The maximal trial was conducted by each swimmer at the center lane of the pool to avoid pacing strategies. The 50 m time trial was recorded by panning one digital video camera (60 fps, GC-IJ20B; Sports Sensing) from the second floor of the indoor pool. The timing when the swimmer's head passed the 15, 25, 35, and 45 m point, which was marked on both side of the lane ropes, and the timing swimmer's hand touched the wall after the starting signal was investigated by feeding the video recording frame by frame at a frame rate of 60 fps. This procedure was repeated three times by the same researcher, and the total time to complete 50 m swim (t50) and the section time of 0–15 m, 15–25 m, 25–35 m, and 35–45 m distances were analyzed.

#### 2.4.2 Load-velocity profile

**Figure 1:**
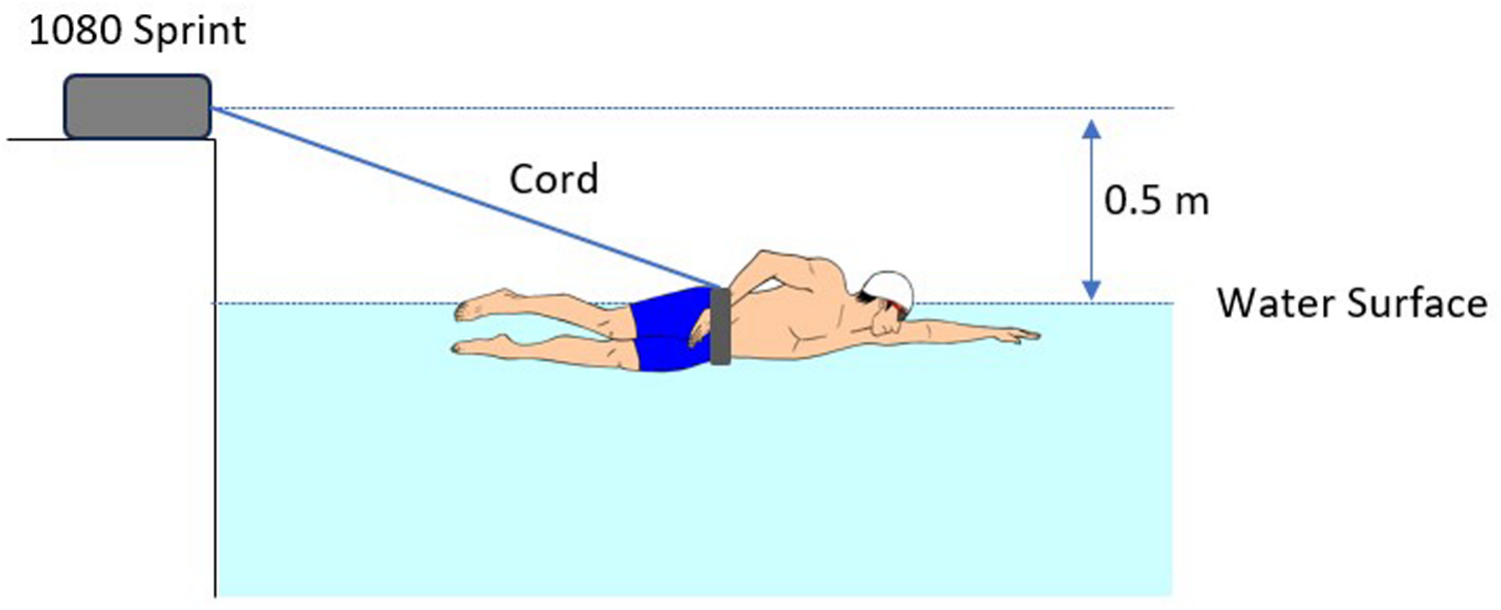
Testing setting for semi-tethered swimming test.

To obtain the load-velocity profile of each participant, a semi-tethered swimming test was performed using a portable robotic resistance device “1080 sprint” (1080 Motion, Lidingö, Sweden). The device was positioned 0.5 m above the water surface on the starting side of the pool (Figure 1). Participants were instructed to wear a swim belt for resisted sprint training around their pelvis (Long Belt Slider S11875, StrechCordz, Bloomington, IL, USA), which was connected to the cord of the resistance device. Each sprint began with a push-off start in the water, and the participants were instructed to start swimming before reaching the 5 m mark. After the warm-up, participants had to perform five 25 m front crawl sprints with maximal effort. There was a rest interval of more than 6 min between each sprint. The instantaneous time-swim velocity data were collected at 333 Hz. The mean velocity during the three stroke cycles between 10 and 20 m section was analyzed using the 1080 sprint software. The velocity measurement cord was not aligned with the swimming direction because the measurement device was placed 0.5 m above the water surface. As a result, the measured velocity was adjusted to the horizontal velocity based on Gonjo et al. (30).

**Figure 2:**
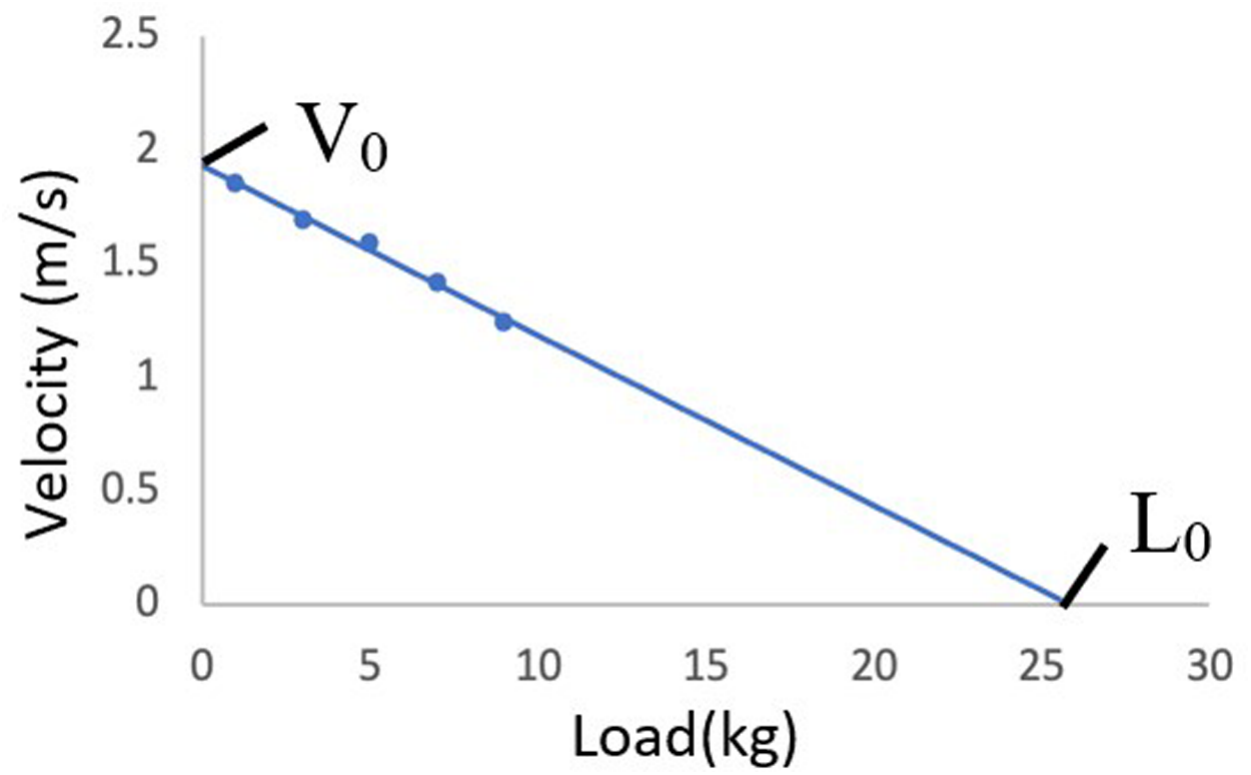
An example of the load-velocity profile measured from one swimmer in this study.

The load-velocity profile of the sprint swim was calculated. The added loads for each sprint were 1, 3, 5, 7, and 9 kg (investigated in ascending order). Olstad et al. (31) reported that 9 kg load imposed more than a 50% velocity reduction compared to a 1 kg load trial in swimmers. This aligns with the suggestion by Cross et al. (32) that a 50% velocity decrease is appropriate for multiple trial sprint testing with external loads. Based on these findings, Gonjo et al. (24) proposed that an absolute load with a maximum of 9 kg is adequate for a semi-tethered swimming test. While Olstad et al. (31) indicated that using five trials (i.e., 1, 3, 5, 7, and 9 kg) in semi-tethered front crawl swimming does not significantly change the outcomes of load-velocity profiling compared to three trials (1, 5, and 9 kg), we opted the five-load protocol to obtain a more precise load-velocity relationship. By examining the linear regression line of the five load-velocity plots (33), the theoretical maximal velocity (V_0_) and maximal load (L_0_) were investigated. L_0_ was also expressed as a percentage of body weight (rL_0_). The slope of the load-velocity relationship was also determined (31). The load-velocity slope indicates the resistive force and is reportedly correlated with active drag in front crawl swimming. A steeper slope indicates lower active drag (34). Figure 2 shows an example of the load-velocity profile measured for the sprint performance parameters.

### 2.5 Statistical analysis

All data are reported as mean ± SD and 95% confidence intervals was calculated. Normal distribution of variables was assessed using the Shapiro–Wilk test. The reliability of ċLa_max_ (measured twice in 11 swimmers) was analyzed using the intra-class correlation coefficient (ICC). ICC was classified as “excellent” (ICC ≥ 0.90), “good” (0.90 > ICC ≥ 0.75), “moderate” (0.75 > ICC ≥ 0.50), or “poor” (ICC < 0.50) (35). The relationships between ċLa_max_ and sprint performance parameters were investigated using Pearson's correlation coefficient for normally distributed data and Spearman's rank correlation coefficient for non-normally distributed data. Correlation threshold values of 0.1, 0.3, 0.5, 0.7, and 0.9 were interpreted as small, moderate, large, very large, and extremely large correlations, respectively (36). The power of the correlation test was also computed. The analysis was performed with SPSS software (version 29, IBM, Tokyo, Japan), and the statistical significance level was set at 5%.

## 3 Results

The measured parameters are shown in Table 2. The average time of the 20 m sprint for the ċLa_max_ measurement was 11.5 ± 0.4 s, La_pre_ was 1.6 ± 0.4 mmol/L, La_max_ was 6.8 ± 1.2 mmol/L, tLa_max_ was 1.8 ± 0.8 min, and ċLa_max_ was 0.63 ± 0.14 mmol/L/s. ICC of the twice-measured ċLa_max_ was 0.913 (95% confidence interval: 0.724–0.976, *p* < .01). ċLa_max_ showed significant correlation with t50 (*r* = −.546, *p* < .05) and the section time of 0–15 m (*r* = −.627, *p* < .01), 15–25 m (*r* = −.618, *p* < .01), 25–35 m (*r* = −.604, *p* < .05) of the trial. L_0_ (*r* = .837, *p* < .01), rL_0_ (*r* = .820, *p* < .01), and the slope (*r* = .804, *p* < .01) obtained from the load-velocity profile also significantly correlated with ċLa_max_.

**Table 2:** C˙la_max_ and its correlation with measured parameters.

			Mean ± SD	95% confidence interval	Correlation between ċLamax	Power of correlation
ċLa_max_		(mmol/L/s)	0.63 ± 0.14	0.56∼0.70		
50 m time trail	0–15 m	(s)	5.93 ± 0.27	5.80∼6.05	-.627**	.817
	15–25 m	(s)	5.18 ± 0.18	5.10∼5.26	-.618**	.796
	25–35 m	(s)	5.31 ± 0.16	5.24∼5.39	-.604*	.771
	35–45 m	(s)	5.41 ± 0.14	5.34∼5.48	-.465	.490
	total time	(s)	24.58 ± 0.73	24.23∼24.93	-.546*	.657
Load-velocity profile	V_0_	(m/s)	1.81 ± 0.09	1.77∼1.86	.224	.139
	L_0_	(kg)	18.56 ± 3.83	16.74∼20.38	.837**	.996
	rL_0_	(%)	25.94 ± 4.79	23.66∼28.21	.820**	.993
	slope	(−m/s/kg)	−0.10 ± 0.02	−0.11 to −0.09	.804**	.989

* *p* < .05, **p < .01.

## 4 Discussion

The present study explored the relationship between ċLa_max_ and factors associated with sprint performance in male competitive swimmers. The main findings of this study were that ċLa_max_ is significantly correlated with 50 m front crawl performance in male swimmers and that a higher glycolytic power contributes to a faster time at the beginning of the sprint race. We were also able to clarify that ċLa_max_ is significantly related to the theoretical maximal load a swimmer can pull with front crawl swimming.

The time taken for the 20 m sprint to measure ċLa_max_ was 11.5 ± 0.4 s, which falls within the recommended range of 10–15 s for this parameter (19). In a study by Langley et al. (37), it was found that the 10-s sprint duration produced the highest ċLa_max_ value when compared to the 15-s and 30-s sprints in cycling tests. Mavroudi et al. (21) also reported higher ċLa_max_ values when investigating shorter sprints in swimming. Additionally, Quittmann et al. (16) suggested that investigating ċLa_max_ based on a fixed distance instead of a fixed duration protocol may be more appropriate in field-based settings if the duration variation is minimal. Overall, the 20 m sprint utilized in this study was appropriate and applicable for assessing a swimmer's highest glycolytic power. The ċLa_max_ value calculated in this study was 0.63 ± 0.14 mmol/L/s, which is similar to the values reported by Sperlich et al. (20) and Mavroudi et al. (21) of 0.64 ± 0.29 and 0.75 ± 0.18 mmol/L/s, respectively. However, in research with young female swimmers, Kellar and Wahl (22) found a lower value of 0.38 ± 0.11 mmol/L/s in their research with young female swimmers. It is worth noting that although ċLa_max_ was assessed using different methods in these studies, it seems that the value is lower in females compared to males, possibly due to differences in muscle mass (38), which could affect lactate production during exercise (39). Generally, female swimmers tend to have longer completion times for a 20 m sprint compared to male swimmers. Additionally, if lactate production is lower in females, ċLamax values will tend to be lower due to the formula used to calculate this parameter (Equation 1). These factors suggest that the relationship between ċLamax and performance may be gender-specific.

The reliability of the ċLa_max_ was “excellent” when measured twice within 4 days (ICC = 0.913). This ICC value was slightly higher than those reported in previous investigations on cycling [ICC = 0.904, (40)], running [ICC = 0.907, (16)], and rowing [ICC = 0.85, (17)]. The accuracy and reliability of the Bla measurement device used in this study were evaluated as good according to the methods of Bonaventura et al. (26). It is worth noting that given the small volume of sampled blood (0.3 µl), we took the average Bla value assessed by the two devices to ensure a stable value. The measurement procedure used in this study clarified that ċLa_max_ could be investigated with high reliability.

Mavroudi et al. (21) studied eight male and four female swimmers and found that a 50 m sprint trial significantly correlated with ċLa_max_ measured during that swim; however, this result may be affected by including both genders. In the present study of male-only measurements and a 20 m sprint, we also found a significant correlation between ċLa_max_ and t50. On the other hand, Kellar and Wahl (22) investigated the relationship between ċLa_max_ and 50 m swim race performance but found no significant relationship. The differences in the measurement protocol may cause different results. Kellar and Wahl (22) investigated ċLa_max_ from a 20-s sprint test in which the duration is longer than the highest value reportedly observed. The sprint duration should be shorter to assess ċLa_max_ related to 50 m performance. Moreover, our measurement setting was defined by the fact that the short sprint was executed from a floating position. This execution eliminates the movement of the block start or wall push and the underwater phase, which is completely different from the regular swimming motion. Thus, this indicated that accurately assessing ċLa_max_ could be achieved by excluding factors other than swimming motion, allowing for the practical evaluation of glycolytic power during swimming. As a result, a 20 m sprint swim solely relying on the swimming motion is an effective way to measure ċLa_max_, making it an important parameter to monitor during training.

When examining the relationship between ċLa_max_ and each section time of the 50 m trial, large correlations were observed at the 0–15 m, 15–25 m, and 25–35 m section times. The contribution ratio of aerobic and anaerobic metabolism is reportedly 30:70 in a 50 m race (1). Therefore, a higher glycolytic power is related to a faster time up to 35 m of the race, while aerobic capacity may be related to the later performance. Therefore, the present study confirmed that ċLa_max_ is a good performance indicator in the first half of a 50 m front crawl swim, suggesting that glycolytic power is crucial for 50 m sprint performance, and swimmers aiming to start fast in a 50 m race should focus on improving ċLa_max_. Research has shown varied effects of different training modalities on ċLamax. Sperlich et al. (20) reported an increase in ċLa_max_ following high-intensity interval training in junior swimmers. Similarly, Nitzsche et al. (41) observed an increase in ċLa_max_ with resistance training in male strength-trained volunteers. However, contrasting results were found by Hommel et al. (14), who reported a decrease in ċLa_max_ after a six-week sprint interval cycling training program. These conflicting findings underscore the need for further research on training interventions specifically aimed at improving glycolytic power in swimmers.

ċLa_max_ correlated with L_0_ and rL_0_, indicating the connection between glycolytic power and the ability to exert force in the water. As Maglischo (2) highlighted, the limiting factors of a 50 m race performance are the stroke technique, the rate of anaerobic metabolism, and the amount of creatine phosphate in the working muscle fibers of a swimmer. Therefore, ċLa_max_ could evaluate the first two factors, making it a valuable parameter for monitoring the swimmer's sprint performance capacity throughout the training season. Coaches and athletes can use ċLa_max_ and aerobic capacity parameter to plan and prescribe training for performance improvement and to avoid nonfunctional overreaching (19, 29). Our study also proposed a practical procedure to evaluate ċLa_max_ in swimming.

A very large correlation was found between ċLa_max_ and L_0_, rL_0_, and the slope calculated from the load-velocity profile. L_0_ represents the theoretical maximal load that swimmers can exert on the water (31). As active drag is approximately proportional to the cube of swimming velocity (42), swimmers need to overcome a large amount of drag during high-velocity swimming. Consequently, a high L_0_ is one of the essential abilities for sprinters. rL_0_ correlates significantly with 50 m front crawl performance (24). Therefore, the present results indicate that the pure capacity to produce power at a higher rate is related to the higher capacity to apply force to the water, which could be crucial for achieving high sprinting performance. Moreover, the significant correlation between ċLa_max_ and the load–velocity slope may be affected by the significant relationship between ċLa_max_ and L_0_. Based on our findings, V_0_ did not correlate with ċLa_max_. As V_0_ represents the theoretical maximal velocity, it may be closely associated with the contribution of the alactic energy system. Furthermore, Gonjo et al. (24) suggested that V_0_ should not be used to predict the absolute free-swimming speed during a race due to the observed systematic bias between the average swimming velocity of the 50 m front crawl trial and V_0_, which may have affected our results.

Beyond evaluating an athlete's glycolytic power, ċLa_max_ has potential applications in predicting individual pacing strategies. Quittmann et al. (43) suggested that this parameter could be used to predict pacing strategies in 5,000 m running, a concept that may be applicable to sprint swimming races. Swimmers with high ċLa_max_ values are likely to start races quickly and apply greater force to the water. For these athletes, coaches should focus on strategies that allow for a fast start while minimizing effort, to prevent fatigue at the end of the race. Conversely, swimmers with lower ċLa_max_ values should avoid an intense burst immediately after the breakout. Instead, they should focus on gradually increasing their effort towards the end of the race. By tailoring race strategies to individual ċLa_max_ profiles, coaches and swimmers can optimize performance and energy distribution throughout the race. Comparing the relationship between ċLa_max_ and race performance across multiple competitions may provide valuable insights into whether success or failure in a race is primarily due to physiological or technical factors. This approach could help coaches and athletes differentiate between improvements in anaerobic capacity and enhancements in swimming technique or race strategy. Furthermore, Morais et al. (44) reported that swimmer with faster critical speed was more likely to perform better in middle- or long-distance events than in sprints. By examining critical speed together, it may provide more detailed information to coaches and swimmers including evaluation of aerobic capacity. Longitudinal studies are warranted to fully clarify the practical applicability of ċLa_max_ in real-world competitive settings. Such research could reveal how this parameter changes over time with training and how these changes correlate with performance improvements. Additionally, it could help establish ċLa_max_ as a reliable tool for long-term athlete monitoring and performance prediction.

A limitation of the present study is that a constant value of 3 s was used for *t*_alac_ to calculate ċLamax. Yang et al. (45) reported that insights into the inter-individual differences in energy and glycolytic metabolism would be altered by using different *t*_alac_ values. An alternative procedure to assess *t*_alac_ is being explored (46). The actual time of *t*_alac_ likely varies among participants, which may influence the precision of ċLa_max_ calculations. If novel approaches to measure power output during swimming can be developed, it would enable a more accurate evaluation of ċLa_max_. Such advancements in measurement techniques could potentially affect the results of the present study and provide more precise insights into the relationship between ċLa_max_ and swimming performance. However, it still seems complicated to establish a rational method to assess t_alac_ during swimming exercise; using a constant value may remain a practical procedure to investigate ċLa_max_ in this sport. Second, Affonso et al. (47) reported an extremely high Bla level of 12.1–17.4 mmol/L after a 5-s short sprint in an elite front crawl swimmer (World Aquatics point = 985). The high ċLa_max_ in this swimmer suggests that higher glycolytic power may be essential for achieving high sprint performance. However, it should be noted that this relationship may have been observed due to the swimmers having identical mechanical efficiencies. Toussaint (48) examined the propelling efficiency of well-trained competitive swimmers and triathletes, reporting values of 61 ± 6% for swimmers and 44 ± 3% for triathletes. This suggests that, even if two swimmers exert the same glycolytic power, a swimmer with lower mechanical efficiency will be slower. It is essential to consider the implications of our study when applied to swimmers at different performance levels. Several methods exist for investigating propulsive efficiency during sprint swimming, including the MAD (Measuring Active Drag) system (48) and pressure distribution analysis (49). By combining these propulsive efficiency evaluations with ċLa_max_ assessments, it would be possible to develop a more comprehensive profile of a swimmer's characteristics. This integrated approach could provide valuable information for planning future training strategies. Specifically, it would allow coaches and athletes to determine whether to focus on improving stroke mechanics or enhancing anaerobic power, based on individual strengths and weaknesses identified through these complementary assessments. Such a multifaceted evaluation strategy could lead to more targeted and effective training interventions, potentially optimizing performance improvements in sprint swimming.

## 5 Conclusion

The testing procedure of ċLa_max_ used in the present study was found to be suitable for training settings and to have high reliability. The study clarified that ċLa_max_ is a parameter associated with the 50 m front crawl performance in male swimmers, and a higher glycolytic power leads to faster times at the beginning of a sprint race. It also indicated that ċLa_max_ can assess a swimmer's ability to apply force to the water during high-intensity swimming. Therefore, ċLa_max_ is a good indicator of front crawl sprint performance for well-trained male swimmers.

## Data Availability

The original contributions presented in the study are included in the article/Supplementary Material, further inquiries can be directed to the corresponding author.
